# Joint Estimation of Source Range and Depth Using a Bottom-Deployed Vertical Line Array in Deep Water

**DOI:** 10.3390/s17061315

**Published:** 2017-06-07

**Authors:** Hui Li, Kunde Yang, Rui Duan, Zhixiong Lei

**Affiliations:** 1 School of Marine Science and Technology, Northwestern Polytechnical University, Xi’an 710072, China; ddstc2008@163.com (H.L.); duanrui@nwpu.edu.cn (R.D.); leizhixiong@163.com (Z.L.); 2 Key Laboratory of Ocean Acoustics and Sensing (Northwestern Polytechnical University), Ministry of Industry and Information Technology, Xi’an 710072, China

**Keywords:** joint localization, vertical line array, reliable acoustic path

## Abstract

This paper presents a joint estimation method of source range and depth using a bottom-deployed vertical line array (VLA). The method utilizes the information on the arrival angle of direct (D) path in space domain and the interference characteristic of D and surface-reflected (SR) paths in frequency domain. The former is related to a ray tracing technique to backpropagate the rays and produces an ambiguity surface of source range. The latter utilizes Lloyd’s mirror principle to obtain an ambiguity surface of source depth. The acoustic transmission duct is the well-known reliable acoustic path (RAP). The ambiguity surface of the combined estimation is a dimensionless ad hoc function. Numerical efficiency and experimental verification show that the proposed method is a good candidate for initial coarse estimation of source position.

## 1. Introduction

The features of reliable acoustic path (RAP) as a stable acoustic transmission duct in the deep ocean have been studied extensively [1,2,3]. The definition of RAP requires a positive critical depth, which specifies that the ocean environment supports long-range propagation without bottom interaction. However, in practice, when the positive critical depth does not exist but the ocean is sufficiently deep, the near-bottom hydrophone can also receive the strong direct (D) signals from the moderate source range. In addition, the interference signals from the long-distance source can also be weakened due to the multireflection with the ocean interface. Generally, such acoustic transmission duct is also called generalized RAP. The present study is under the generalized RAP environment.

Under RAP environment conditions, our previous work proposed two single-hydrophone localization methods for a moving radial source [4,5] and a two-hydrophone localization method with cross-correlation function matching [6]. Nevertheless, these methods commonly require that the signal-to-noise ratio (SNR) is higher than 0 dB. McCargar et al. [7] introduced a source depth estimation method with a vertical line array (VLA) by exploiting the coherent addition structure of the D and surface-reflected (SR) arrivals; Kniffin et al. investigated the performance metrics of this method [8]. The method also requires long duration for observation, and it is vulnerable to ocean fluctuation. All these methods are associated with the multipath arrival structure at the receiver hydrophone or array. Specifically, research only focused on the D and SR arrivals because the bottom-reflected (BR) arrivals are often not available due to the weak arrival amplitude and considerable spread in time. Other relevant studies related with source localization by employing the information of multipath arrival structure are reported in Refs. [9,10,11,12] and references therein.

The previous methods commonly do not work well for passive detection in actual ocean environments due to the lack of available stable information. In practice, the VLA is a common receiving geometry, and it can sufficiently sample the acoustic field information for source localization. For example, matched filed processing (MFP) is a typical generalized beamforming method that uses spatial complexities of acoustic fields to localize source [13]. Generally, the signals received by a VLA include three kinds of source information: direction of arrival (DOA) in space domain, time delay in time domain, and interference characteristic in frequency domain. Any of them cannot accurately localize the source alone. According to the ray propagation point of view, the ray paths under RAP environment are clear and stable. The distinct property of RAP provides very reliable information for source localization. On the basis of the space-frequency features of RAP, we propose a joint source localization method in this paper. We utilize the spatial information in the form of arrival angle of the D path and the frequency-dependent interference characteristic between the D and SR paths; the former is based on a ray backpropagation procedure, and the latter is related to Lloyd-mirror effect [14]. The advantages of the proposed method include the sufficient utilization of the array aperture to estimate the DOA of the D path and the high output SNR after beamforming, which enhances the interference characteristics in frequency domain. Finally, a dimensionless range/depth ambiguity surface is provided. The localization results are robust. Moreover, the beamforming technique can also potentially suppress the interference source outside the main lobe in complex environments. In Section 2, a detailed description of the method and the simulation results are given. In Section 3, the proposed method is verified using experimental data. Localization errors are discussed in Section 4. Section 5 summarizes this paper.

## 2. Theory and Simulation

**Figure 1:**
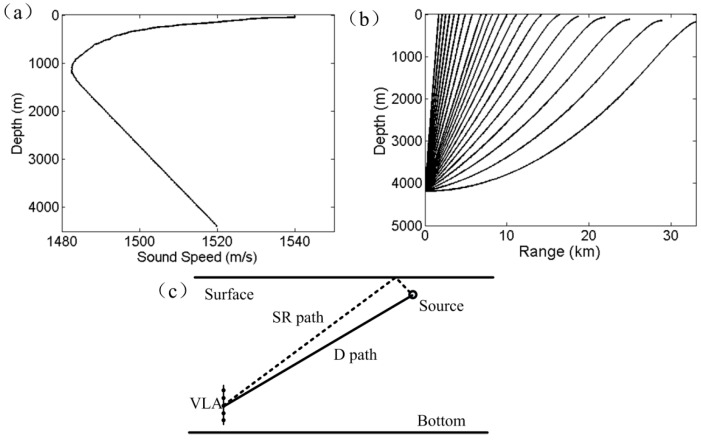
(**a**) Sound speed profile from an experimental measurement. (**b**) Ray propagation with source depth of 4188 m. (**c**) Geometry for Lloyd’s mirror effect.

The source localization method consists of three steps. The first step uses the VLA to estimate the DOA of the source signal using conventional beamforming (CBF). Figure 1a shows the sound speed profile (SSP) used in numerical simulation, this profile was obtained from an experimental measurement that will be introduced in Section 3. The rays emitting from a source at 4188 m are presented in Figure 1b. The ray bending effect obeys Snell’s law. When the arrival angle of the D path is obtained by CBF, the stable ray model is used to trace the possible source locations and obtain a range ambiguity surface. The second step utilizes the interference characteristic of beamforming output. The geometry of Lloyd’s mirror effect is illustrated in Figure 1c. 

The beamforming output contains the information about D and SR arrivals, the interference oscillation of which along frequency is used to obtain a depth ambiguity surface. The last step combines the above two results to obtain a dimensionless ambiguity surface with the maximum value corresponding to the estimated source location.

### 2.1 Range Estimation Method

The implementation of range estimation is based on an assumption that the estimated DOA by CBF indicates the arrival angle of D path. The frequency-incoherent broadband output power of CBF is as follows [15]:

(1) $$P\left(\theta \right)=\frac{1}{L}\sum_{l=1}^{L}w^{H}\left(f_{l},\theta \right)R\left(f_{l}\right)w\left(f_{l},\theta \right)$$

where *L* is the number of frequency bins in the frequency band of interest, ***w*** is the weight vector of CBF and a function of frequency *f_l_*and look direction *θ*, superscript H denotes the conjugate transpose of a vector, and ***R*** is the cross-spectral density matrix of the receiver signals and a function of frequency *f_l_*. The weight vector is given as follows:

(2) $$w(f_{l},\theta )={\left[e^{-j(\frac{N-1}{2})kd\cos\theta },e^{-j(\frac{N-1}{2}-1)kd\cos\theta },\cdots ,e^{j(\frac{N-1}{2}-1)kd\cos\theta },e^{j(\frac{N-1}{2})kd\cos\theta }\right]}^{T}$$

where *N* is the number of hydrophones, *d* is the hydrophone spacing, superscript T denotes the transpose of a vector, and wavenumber *k* = 2π*f_l_*/*c*_CBF_, where *c*_CBF_ is the reference sound speed. The reference sound speed should be the averaged sound transmission speed connecting the source to the receiver. In this paper, *c*_CBF_ is the sound speed at the midpoint receiver depth of VLA. 

For simplicity, we assume the array signals only coming from a near-surface source of interest. Thus, the maximum of *P*(*θ*) corresponds to the estimated arrival angle of D path, that is:

(3) $$\theta_{0}=\underset{\theta }{\arg\max}\;P\left(\theta \right)$$

When the source of interest is submerged by strong interference, further technology is required to determine the arrival angle of the D path from the source of interest. 

The hypothesized source location is denoted by *L_h_* = [*z, r*], where *z* is the source depth and *r* is the horizontal range from the VLA. The arrival angle of D path will be a function of the hypothesized source location. This function can be expressed as follows:

(4) $$\theta_{h}=g\left(L_{h}\right),$$

where *g*(.) is determined by the acoustic environment, and it can be calculated by using a standard ray approach. The candidate for source location is at the range-depth grid with minimum difference between *θ*_0_ and *θ_h_*. The ambiguity surface is defined as follows:

(5) $$E_{1}=-\left|\theta_{0}-\theta_{h}\right|$$

when the hypothesized source localization is the same with real source localization, *E*_1_ is the maximum and nearly equal to 0°.

**Figure 2:**
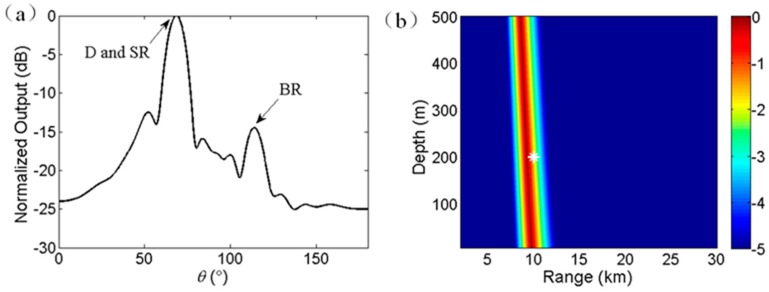
(**a**) Norlimalized output power of CBF for the source at 200 m depth and 10 km range. (**b**) Ambiguity surface for range estimation with *θ*_0_ = 68.8°.

A numerical example is presented to illustrate the range estimation approach. The simulation environment follows the experiment scene. The SSP is shown in Figure 1a with ocean depth of 4390 m. Besides, range independence is also assumed. The source depth is 200 m, the receiver depth at the midpoint of the VLA is 4188 m, and the horizontal range is 10 km. The VLA consists of 16 hydrophones spaced 4 m apart. The source signal is generated by filtering the white Gaussian noise with a bandpass filter. The frequency band of the signal is 100–200 Hz to avoid the occurrence of grating lobe. The broadband signal is uncorrelated across frequencies. The array signals are simulated by a standard ray approach [16]. Figure 2a shows the normalized output power of CBF, whose peaks indicate the multipath arrival angles. The maximum power occurs at 68.8°, which corresponds to the arrival angle of D path. The D and SR arrivals cannot be resolved on the basis that the difference of D and SR arrival angles is too small to distinguish for CBF. The second peak at 113.7° is due to the arrivals related with BR, whose intensity is affected by bottom parameters. Although CBF is not an optimal choice in distinguishing all the multipath arrivals, the range estimation error induced by the angle estimation error (measurement error) is tolerated in our current study. Moreover, CBF exhibits a wide main lobe that allows the simultaneous beamforming output of D and SR paths, which is also necessary for the depth estimation in the next subsection.

The ambiguity surface using Equation (5) is shown in Figure 2b, where the real source location is denoted by a white asterisk. The unit of the ambiguity surface is degree. The ambiguity surface presents sloping straight striation across the real source location. The average arrival angle of D and SR paths is weakly dependent on source depth [17]. Thus, Equation (5) can only provide a preliminary estimation of source range, but it fails to estimate the source depth.

### 2.2 Depth Estimation Scheme

The received array signal is assumed as *x*(*t*). When the estimation of arrival angle *θ*_0_ is completed, the time series *y*(*t*) is subsequently obtained after broadband CBF steering at *θ*_0_. According to image theory, the complex acoustic pressure *g*(*f*), the Fourier transform of *y*(*t*), are oscillating with frequency, and the oscillatory period is associated with the source location. The amplitude variation of *g*(*f*) takes the following simple form [14]:

(6) $$\left|g\left(f\right)\right|=\frac{2}{R}\left|\sin\left(\frac{2\pi f}{c}z_{s}\sin\varphi \right)\right|,$$

where $R=\sqrt{{z_{r}}^{2}+r^{2}}$, *c* is the sound speed for the ideal constant SSP, *z_s_* is the source depth, and $\varphi =\arctan\left(z_{r}/r\right)$. The sinusoidal structure in Equation (6) results from the constructive or destructive addition of D and SR arrivals, whose periodicity is strongly related with the source depth and source range. The sinusoidal modulation exhibits periods of π, which yields the following:

(7) $$z_{s}=\frac{c}{2\Delta f\sin\varphi },$$

where Δ*f* is the width of the modulation between nulls. The interference pattern can be transformed into source depth information by the Fourier transform scheme [18,19]. The process involves the following steps: (a) obtaining of the time series *y*(*t*) by CBF, (b) Fourier transforming *y*(*t*) to frequency domain *g*(*f*), and (c) Fourier transforming *g*(*f*) to the depth and range domain:

(8) $$x\left(t\right)\underset{\;\mathrm{CBF}}{\;\to }\;y\left(t\right)\;\underset{fft}{\to }\;g\left(f\right)\;\underset{fft}{\to }\;h\left(z_{s},r\right)$$

According to Equation (8), the final step involves two variables, namely, *c* and *φ*. *c* is the sound speed at the receiver depth *z_r_*, and *φ* relies on the search range *r*. For different *r* values, Δ*f* indicates different source depth *z_s_*. Hence, the ambiguity surface *h*(*z_s_*,*r*) will be a function of search range and search depth.

**Figure 3:**
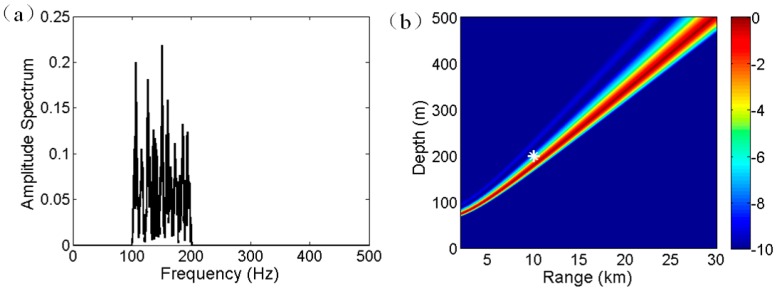
(**a**) Amplitude spectrum of *g*(*f*) in frequency domain. (**b**) Ambiguity surface for depth estimation.

For the considered example, Figure 3a shows the amplitude spectrum *g*(*f*) of time series *y*(*t*) after Fourier transform. The frequency band of interest ranges from 100 to 200 Hz. As illustrated in Equation (6), the amplitude (energy) oscillates along frequency, whose oscillatory period (null width) reflects the source location, as given in Equation (7). The Fourier transform of the amplitude spectrum of *g*(*f*) for each search range provides an estimation of source depth. In detail, after Fourier transform, frequency bins are converted into source depth bins by multiplying by *c*/2/sin*φ* [5]. Notably, the depth resolution varies with the search range. In the present study, interpolation is adopted to obtain the estimated value at an arbitrary source depth. The normalized ambiguity surface for depth estimation in dB is defined as:

(9) $$E_{2}=10\;\log_{10}\left(\frac{h\left(z_{s},r\right)}{\max(h\left(z_{s},r\right))}\right)$$

According to Equation (9) the ambiguity surface is calculated and shown in Figure 3b, where the real source location is denoted by a white asterisk. The unit of the ambiguity surface is dB. The ambiguity striation passes the real source location. In addition, in any real source range, the source is most likely a submerged one due to that the estimated source depth is larger than the common depth of a surface source [20]. Consequently, the interference characteristic of D and SR paths can be used for source depth discrimination even for a single hydrophone, given that the SNR is sufficiently high. 

### 2.3 Joint Estimation of Source Range and Depth 

Neither the arrival angle of D path in space domain nor the interference characteristic of D and SR paths in frequency domain has yet given a sole estimation of source location. However, the source location can be estimated accurately by combining the two methods described. The ambiguity surface of the joint method is given as follows:

(10) $$E_{C}=\varepsilon \frac{E_{1}}{\left|m\left(E_{1}\right)\right|}+\frac{E_{2}}{\left|m\left(E_{2}\right)\right|}$$

where $\left|m\left(E_{1}\right)\right|$ and $\left|m\left(E_{2}\right)\right|$ are the absolute values of the averages of *E*_1_ and *E*_2_, respectively, and *ε* is an adjusting factor to balance the dynamic range of *E*_1_ and *E*_2_. In this paper, *ε* is given as follows:

(11) $$\varepsilon =\left|\frac{\min\left(E_{2}\right)}{\min\left(E_{1}\right)}\right|$$

The existence of *ε* will not cause any variation to the estimation results, but will provide a good demonstration. Equation (10) is an ad hoc cost function, and *E_C_* is dimensionless. The maximum in the ambiguity surface indicates the estimated source location. 

**Figure 4:**
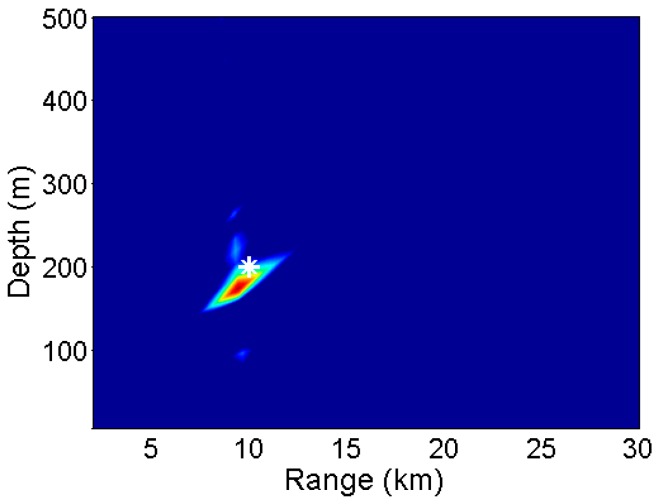
Ambiguity surface of joint estimation of source depth and source range. The real source location is at 10 km range and 200 m depth. The estimated source location is at 9.7 km range and 183 m depth.

For the numerical example, the joint estimation of source location using Equation (11) is shown in Figure 4. The range and depth search intervals are 0.1 km and 1 m, respectively. The estimated source range and source depth are 9.7 km and 183 m, respectively. The estimated location is largely close to the real source location. Furthermore, the colorbar disappears because the estimation results are dimensionless, and it shows no significant meaning. The localization errors will be discussed in Section 4.

### 2.4 Effects of SNR

In our previous study [4], the received SNR on one hydrophone influences the striation identification of D-SR time delays in time domain. From the frequency domain point of view, the interference characteristic of D and SR paths is also related with the SNR. The effects of SNR on source estimation are discussed in this Subsection. 

Noise is generated the same way as signal. The noise level is fixed. Thus, the input SNR on one hydrophone decreases with the decreasing source level. The SNR is defined as the average signal power in the signal bandwidth divided by the average noise power in the same bandwidth. The SNR increases from −25 dB to 10 dB. The source is assumed at 10 km range and 200 m depth. The search range is 10 km. The source depth estimation results are quantitatively calculated both using single hydrophone and the whole VLA. The depth estimation scheme and other simulation conditions follow the description in Section 2.2.

**Figure 5:**
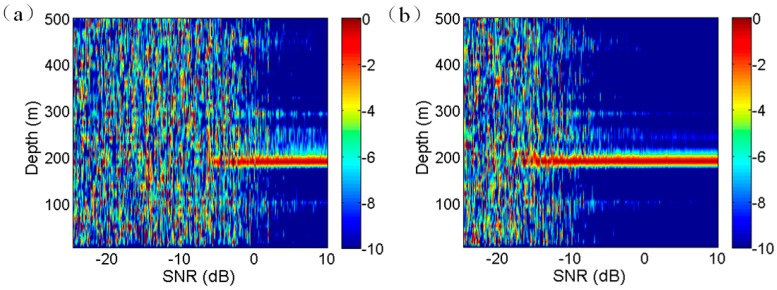
Comparison of the effects of SNR on depth estimation by using (**a**) single hydrophone and (**b**) the whole VLA.

The depth estimation results using single hydrophone for different SNRs are shown in Figure 5a. When the SNR is lower than approximately –5 dB, the estimation results are blurry, and stable estimation values cannot be obtained. As a contrast, the depth estimation results obtained by using the broadband beamforming output steering at 68.8° are shown in Figure 5b. Clearly, the least SNR for unambiguous depth estimation is low at about −15 dB. Hence, the VLA offers approximately 10 dB gain. The nominal white noise gain for a 16-element array is 12 dB, the 2 dB array gain degradation (AGD) is due to the multipath interference effect.

Consequently, for the VLA used in this study, as long as the input SNR on one hydrophone is higher than −15 dB, the interference characteristic of D and SR paths can be used for depth estimation.

In addition, increasing the array length can improve the array gain (AG). However, the AG is also closely related to the vertical coherence of the acoustic field at the array receiver depth. The coherence loss of the acoustic field will cause AGD for large aperture array. Besides, increasing the array length may cause other issues, for example, the D and SR paths are not in the same beam of the beamforming output. The further discussion on array size is beyond the scope of this paper.

## 3. Experimental Verification

The localization method was validated through an experiment. The experiment was performed in the South China Sea. In the experimental area, the ocean bottom is almost flat and the ocean depth is 4390 m. A VLA of 16 hydrophones spaced at 4 m was deployed near the ocean bottom. The shallowest hydrophone was 4158 m. The measured SSP at the array location is shown in Figure 1a. The acoustic signals were obtained from the measurements of the sound exposure levels of explosive charges. The receiver range was between 17 and 19 km. The mass of trinitrotoluene (TNT) in the explosive charges was 100 g. Two kinds of explosive sources were used, and the nominal explosive depths were 50 and 300 m. 

**Figure 6:**
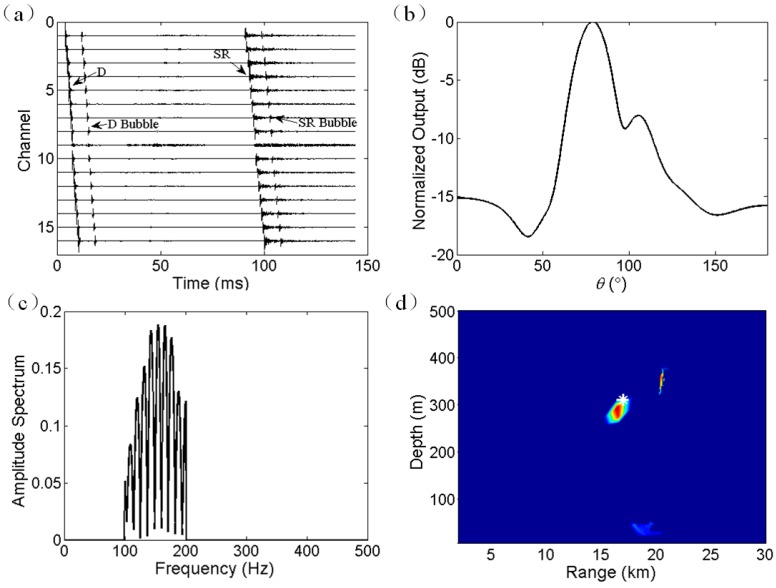
Experiment results for a 300 m explosive charge. (**a**) Received multipath signals on all the hydrophones. (**b**) DOA estimation using broadband CBF with frequency band ranging from 100 to 200 Hz. (**c**) Amplitude spectra of the time series of beamforming output in frequency domain. The frequency band of interest is limited between 100 and 200 Hz. (**d**) Ambiguity surface of joint estimation of source depth and source range. The real source location is at 17 km range and 310 m depth. The estimated source location is at 16.5 km range and 293 m depth.

The waveforms from a 300 m explosive source are shown in Figure 6a. The multipath arrival structure was clear and the time delays among hydrophones were also evident. The positions of bubble pulses were dependent on the source depth and the TNT weight [21]. Although the bubble pulses were evident, these pulses are not eliminated in time domain because no distinct degradation occurred in the localization performance. In addition, the signal on the ninth hydrophone was abnormal and removed in the following analysis. The length of the extracted signal is 1 s. The estimation of DOA by using the broadband CBF is shown in Figure 6b. The frequency band of interest is limited in the range of 100–200 Hz to avoid the occurrence and effect of grating lobes. As shown in Figure 6b, the estimated source bearing is located at *θ*_0_ = 78.7°. In practice, when the source is a weak target, and the received array signals are polluted by other interferences, Equation (3) is no longer applicable, and the arrival angle of D path should be identified by other means. The amplitude spectra of beamforming output in frequency domain is shown in Figure 6c. The frequency band is limited between 100 and 200 Hz. Oscillatory features are evident, as predicated by Equation (6). The joint localization result by using Equation (11) is shown in Figure 6d. The peak appears at a range of 16.5 km and depth of 293 m. According to the time delays of bubble pulses, the actual trigger depth of explosive charge was approximately 310 m [21]. According to the GPS recording, the actual source range was about 17 km. Consequently, the range and depth estimation are biased by about 3% and 5%, respectively.

**Figure 7:**
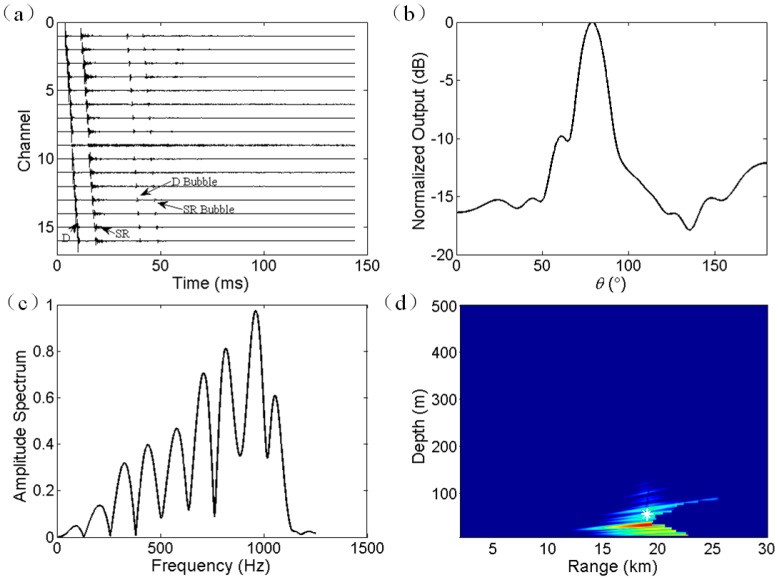
Experiment results for a 50 m explosive charge. (**a**) Received multipath signals on all the hydrophones. (**b**) DOA estimation using broadband CBF with frequency band ranging from 100 to 200 Hz. (**c**) Amplitude spectra of the time series of beamforming output in frequency domain. (**d**) Ambiguity surface of joint estimation of source depth and source range. The real source location is at 19 km range and 55 m depth. The estimated source location is at 18.5 km range and 35 m depth.

The processing approach and results for a 50 m explosive source are shown in Figure 7. Two differences are illustrated as follows. First, as shown in Figure 7a, the bubble pulse arrivals are after D and SR arrivals. Second, the interference period in frequency domain is large, as shown in Figure 7c, due to the shallow explosive depth. For the depth estimation, the frequency band ranging from 100 to 1000 Hz is used. In the ambiguity surface, the peak appears at a range of 18.5 km and depth of 35 m. According to the time delays of bubble pulses, the actual trigger depth of explosive charge was approximately 55 m. According to the GPS recording, the actual source range was about 19 km. Consequently, the range and depth estimation are biased by about 3% and 36%, respectively. Therefore, the depth estimation error is not tolerated. The depth estimation is restricted by the applicability of Lloyd’s mirror principle. The localization error and the applicability of the proposed method will be discussed in the next section.

## 4. Performance Metrics 

This section discusses the localization errors of the proposed method. Errors are the cumulative results of range and depth estimation errors.

First, the range estimation errors are the cumulative results of modeling and measurement errors. Modeling errors are mainly caused by the assumption that the maximum of the beamforming power output corresponds to the arrival angle of D path. In fact, the maximum is the comprehensive result of the D and SR arrival angles. The arrival angel of SR path is smaller than that of the D path. The deep source depth results in large difference between those two angles. Thus, the estimated arrival angel *θ*_0_ by CBF is commonly smaller than the real one of D path. Consequently, the estimated source range by ray backpropagation will be smaller than the real value. At ranges larger than 20 km, the D path will not arrive at the VLA for the source shallower than 100 m. Therefore, Table 1 only provides a list of some simulated localization results for different source-receiver geometries with source range shorter than 20 km. The estimated source ranges for all kinds of source-receiver geometries are smaller than the theoretical values. The modeling errors also include the accuracy of the SSP used in the ray approach. However, the SSP in deep water is relatively stable, especially for RAP environment. The error introduced by the bias of SSP can be ignored in most cases.

Other important factors on range localization error include the measurement errors caused by the selection of reference sound speed *c*_CBF_ in CBF, array tilt, and ocean fluctuation, etc. *c*_CBF_ should be the averaged sound transmission speed connecting the source to the receiver. In our study, the sound speed at the receiver depth is appropriate, and it introduces little DOA estimation error. The array tilt is negligible in our experiment due to its short length.

**Figure 8:**
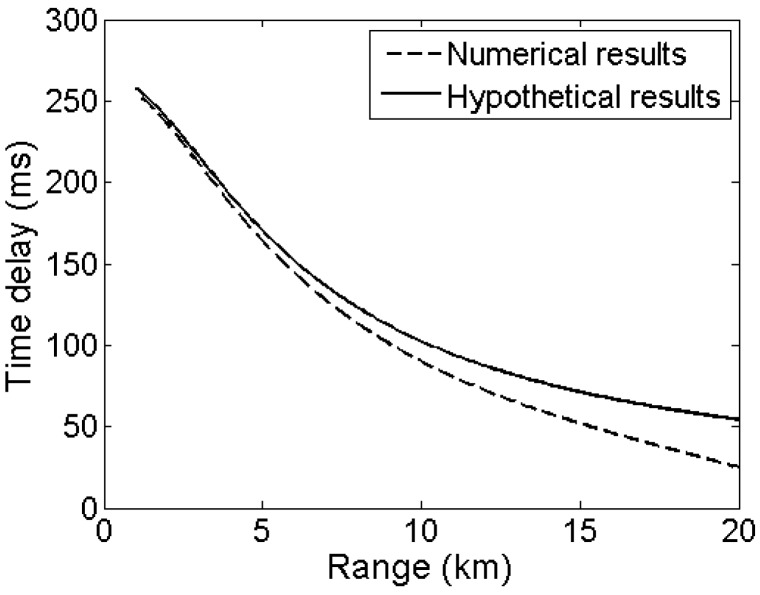
Comparison of numerical and hypothetical results of time delays between D and SR arrivals. The receiver and source depths are 4188 and 200 m, respectively.

Second, the depth estimation errors are also the cumulative results of modeling and measurement errors. Modeling errors are caused by the discrepancies between the ideal Lloyd’s-mirror interference and the actual acoustic field interference. The interference is caused by the constructive and destructive additions of D and SR paths, which are attributed to the difference in time delay of D and SR arrivals. Figure 8 shows the simulated time delays between D and SR arrivals versus range with the dashed line. The source depth and receiver depth are 200 and 4188 m, respectively. The ideal curve of time delay between D and SR arrivals is depicted by the solid line, where the sound speed is constant (1510 m/s). The differences between these two kinds of time delays are gradually increasing with the increase in range. Moreover, the numerical results are smaller than the hypothetical ones. This result implies that at long ranges, the estimated source depths are smaller than the real values; this result is explained by that the shallow source depth results in the small the time delay between D and SR arrivals. As shown in Table 1, at the range of 20 km, the estimated source depths are considerably smaller than the theoretical ones, and they are invalid. The essence is that Lloyd’s mirror effect requires a constant SSP with no ray bending, which does not exist in deep ocean environment. Consequently, the depth estimation is invalid for the source at long ranges.

Another reason that introduces depth estimation is the measurement error, which is implied in the model error. In accordance with Equation (7), the sound speed *c* should be selected manually. We use a sound speed of 1510 m/s, which corresponds to the sound speed at the receiver depth. Different setups of sound speed used in Equation (7) will cause different error variations. For example, when the sound speed is sufficient large to make the hypothetical curve in Figure 8 go below the numerical one, the estimated source depth will be larger than the real ones.

Finally, it is worth mentioning that the range and depth estimation errors show no significant dependence on source depth. 

## 5. Conclusions 

A joint source localization method separating the range and depth estimations apart is proposed in this paper. Two kinds of time-delay information are used. The first one is the multipath time delays among hydrophones, this information is used for range estimation by estimating the arrival angle of D arrival in space domain. The other kind is the time delays between D and SR arrivals, such information is used for depth estimation by estimating the interference period in frequency domain.

The proposed method sufficiently exploits the array aperture to improve the output SNR and enhance the interference characteristic of D and SR paths for depth estimation. In addition, the beamforming technique can also suppress the interference information outside the main lobe in multiobjective scenes. However, at long ranges, Lloyd’s mirror principle does not work well, and the depth estimation error is large. Experimental results using explosive sources exhibit acceptable localization results. The proposed method can be used for the bottom-deployed VLA for underwater autonomous unmanned alerting due to its operation simplification and performance stability for near-range source localization.

## Figures and Tables

**Table 1:** Numerical simulation results for different source positions using the proposed method.

Theoretical Value		Estimated Value	
Range (km)	Depth (m)	Range (km)	Depth (m)
5	100	4.9	97
	200	4.8	196
	300	4.7	295
10	100	9.9	90
	200	9.7	183
	300	9.7	289
15	100	14.9	85
	200	14.8	180
	300	14.7	280
20	100	19.8	50 *
	200	19.8	145 *
	300	19.7	255 *

* indicate that the estimated source depths are doubtful due to the big model error.
